# Applying a computer-aided scheme to detect a new radiographic image marker for prediction of chemotherapy outcome

**DOI:** 10.1186/s12880-016-0157-5

**Published:** 2016-08-31

**Authors:** Yunzhi Wang, Yuchen Qiu, Theresa Thai, Kathleen Moore, Hong Liu, Bin Zheng

**Affiliations:** 1 School of Electrical and Computer Engineering, University of Oklahoma, Norman, OK 73019 USA; 2 Health Science Center of University of Oklahoma, Oklahoma City, OK 73104 USA

**Keywords:** Computer-aided detection (CAD), Quantitative image feature analysis, Prediction of chemotherapy outcome, Clinical image markers for cancer prognosis prediction

## Abstract

**Background:**

To investigate the feasibility of automated segmentation of visceral and subcutaneous fat areas from computed tomography (CT) images of ovarian cancer patients and applying the computed adiposity-related image features to predict chemotherapy outcome.

**Methods:**

A computerized image processing scheme was developed to segment visceral and subcutaneous fat areas, and compute adiposity-related image features. Then, logistic regression models were applied to analyze association between the scheme-generated assessment scores and progression-free survival (PFS) of patients using a leave-one-case-out cross-validation method and a dataset involving 32 patients.

**Results:**

The correlation coefficients between automated and radiologist’s manual segmentation of visceral and subcutaneous fat areas were 0.76 and 0.89, respectively. The scheme-generated prediction scores using adiposity-related radiographic image features significantly associated with patients’ PFS (*p <* 0.01).

**Conclusion:**

Using a computerized scheme enables to more efficiently and robustly segment visceral and subcutaneous fat areas. The computed adiposity-related image features also have potential to improve accuracy in predicting chemotherapy outcome.

## Background

Due to the cancer heterogeneity, patient response to a specific therapeutic treatment varies significantly. In order to improve cancer treatment efficacy, the recent Precision Medicine Initiative calls for developing a new cancer treatment strategy that takes individual variability into account [1]. This requires using effective biomarkers to more accurately characterize patients and/or predict clinical outcome of the patients in participation of the targeted chemotherapy. Although many cancer genomic biomarkers have been discovered [2], which aim to optimally select the targeted therapies to treat cancer patients [3], using existing biomarkers to determine a specific therapeutic treatment strategy for individual patients remains a clinically difficult task because 1) many biomarkers are only applicable to a small group of patients [4, 5], 2) genomic tests are often invasive and expensive [6] and 3) most genomic biomarkers have lower specificity [7]. Therefore, radiographic imaging tests are still important in cancer diagnosis and prognosis assessment. However, reading and interpreting medical images in the clinical imaging facilities has several limitations including the large inter-reader variability and the lack of methods to quantitatively assess useful image features.

In order to overcome these limitations, developing new quantitative image feature analysis methods to increase the discriminatory power in predicting cancer risk and prognosis has attracted wide research interest and efforts recently [8–10]. For example, using the new concept of “Radiomics,” some researchers believed that one can use the quantitative image features computed from medical images including computed tomography (CT) and magnetic resonance imaging (MRI) to build new predictive models to phenotype gene-protein signatures and/or genomic biomarkers. As a result, using quantitative feature analysis has potential to produce new clinical markers to better assist cancer diagnosis and prognosis assessment [11, 12]. However, a prerequisite of developing a reliable quantitative image feature analysis approach is developing an accurate automated image segmentation scheme, which remains a challenging task. Although researchers have previously developed and tested many different computerized schemes to detect and segment different types of suspicious lesions or anatomic structures depicting on different types of medical images, selecting and/or applying which segmentation algorithms depends on the specific application tasks.

Recently, much research effort has been spent on the development of image processing based clinical decision support systems. For example, we previously investigated the feasibility of applying quantitative image feature analysis methods to identify image markers for assisting prediction of prognosis and treatment efficacy of several different types of cancers, which include breast [13], lung [14], and ovarian cancer patients [15, 16]; Ramirez et al. tested an image parameter selection and support vector machine based framework for improving early detection of Alzheimer’s disease [17]; Olsen et al. developed an image-processing based system to enable dental caries detection [18]. In existing image based decision support systems for prognosis and treatment assessment of ovarian cancer, all of the previous studies only computed image features from the segmented tumors from either CT or MR images. For example, Qiu et al. extracted image features related to tumor volume, density and variance for predicting clinical benefit of treating ovarian cancer at early stage [15]; Tan et al. applied a B-spline based image registration process to identify or track tumor changes and assess treatment response [16]. However, besides tumor-related image features, the patients’ overall health condition and other non-tumor related image features may also be important to indicate how the patients will respond to (or receive benefit or not from) the chemotherapy. For example, angiogenesis played a fundamental role in the pathogenesis of epithelial ovarian cancer (EOC) with higher vascular endothelial growth factor (VEGF) expression. It promotes tumor growth, ascites and metastasis [19]. Thus, a bevacizumab-based therapy that targets the angiogenesis-specific pathways has been developed and tested to treat EOC patients in the clinical trials [20–22], which indicated that some EOC patients received benefit with increased progression-free survival (PFS) or overall survival (OS) [21], while some others did not receive any benefit with shorter PFS and OS [20]. Because of the high toxicity and other harmful side effects of using bevacizumab-based chemotherapy [23], it is important to rationally select who are most likely to receive the benefit from bevacizumab or other antiangiogenic therapies among the EOC patients [24]. Among the image feature based clinical markers, a recent study has shown that the ratio of visceral fat areas (VFA) and subcutaneous fat areas (SFA) has been recognized as potentially useful features to predict clinical outcome of bevacizumab-based chemotherapy [25]. However, manually segmenting VFA and SFA in a large number of CT image slices is quite tedious and often inconsistent due to the intra- and inter-reader variability.

In order to overcome the difficulty of manual segmentation and improve consistency and efficiency of SFA and VFA segmentation, we proposed and developed a new computer-aided image feature analysis scheme to automatically segment and quantify the entire volume of the VFA and SFA computed from the abdominal CT images of the EOC patients. We compared the correlation between the fat areas (VFA and SFA) segmented by the automated scheme on volumetric CT image data and a radiologist on one selected CT image slice of each testing case. In addition, to test the potential clinical utility of applying this automated scheme to predict prognosis or treatment efficacy of the EOC patients based on new quantitative image feature analysis method, we also built two logistic regression models using the image features computed from the segmented VFA and SFA and assess the feasibility of applying the new computerized scheme to identify and/or select EOC patients who are most likely to get benefit from receiving the bevacizumab-based chemotherapy.

## Methods

In this study, a retrospectively collected image dataset that involves CT images of 32 patients was assembled based on our Institutional Review Board (IRB) approved data collection and study protocol. All patients were diagnosed and treated with advanced Stage III and IV epithelial ovarian cancer (EOC) in our University Medical Center. For each patient, the contrast enhanced perfusion CT image scans were performed post the primary cyto-reductive surgery and prior to the chemotherapy initiation. All the perfusion CT images were scanned and produced using a GE LightSpeed VCT 64-detector or a GE Discovery 600 16-detector CT machine. As an established testing protocol in our University Medical Center, X-ray power output of the CT machine was set at 120 kVp and a variable range from 100 to 600 mA depending on body size of the patient. In each examination, 100 cc contrast agent of Isovue 370 was intravenously injected using a standard power injector with a rate of 2-3 cc/second before starting CT image scanning.

As a standard procedure, each patient also received chemotherapy of bevacizumab (175 mg/kg) plus paclitaxel (175 mg/m2), and plus carboplatin AUC 6 with a follow-up maintenance bevacizumab. In addition, we collected the PFS and OS data of all 32 patients in the dataset. Among these patients, mean age is 59.5 years old, mean weight is 70 kg along with a mean body mass index (BMI) of 26.1, and the median PFS and OS were 28.9 and 40.8 months, respectively.

**Fig. 1:**
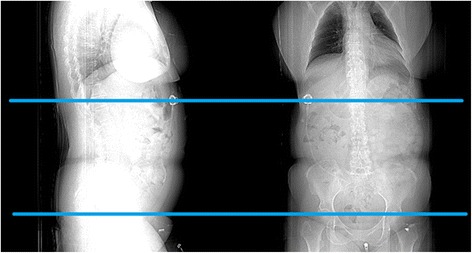
Illustration of CT image scanning range (between two horizontal bars) selected by the CAD scheme to segment VFA and SFA and compute the corresponding image features

For each of the EOC patients, perfusion CT was performed to scan from lung to pelvis, which crosses the entire abdomen region (as illustrated in Fig. 1). In each case, an upper and a lower boundary were manually placed to mark and select a CT scanning range in the entire abdomen scanning region. The upper bound of the selected CT image slices is just located below the lung area, while the lower bound of the region is just placed above the umbilicus level of a patient.

**Fig. 2:**
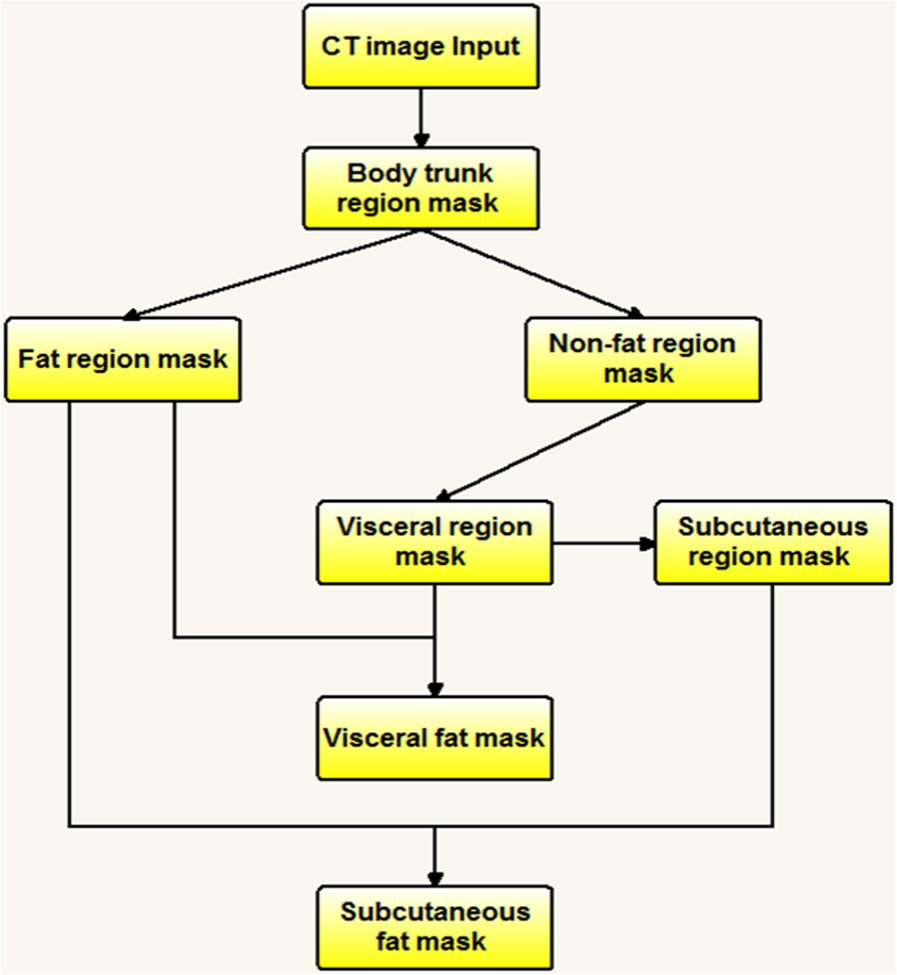
A flowchart of a CAD scheme for segmenting SFA and VFA on CT images

We then developed and applied a computer-aided image processing scheme to (1) detect and segment visceral fat area (VFA) and subcutaneous fat area (SFA) on the masked or selected abdominal CT image slices, and (2) compute volumes of VFA and SFA, as well as the fat density distribution related image features. Figure 2 shows a flow diagram of our computer-aided scheme in detecting and segmenting VFA and SFA with the following four image processing steps.

**Fig. 3:**
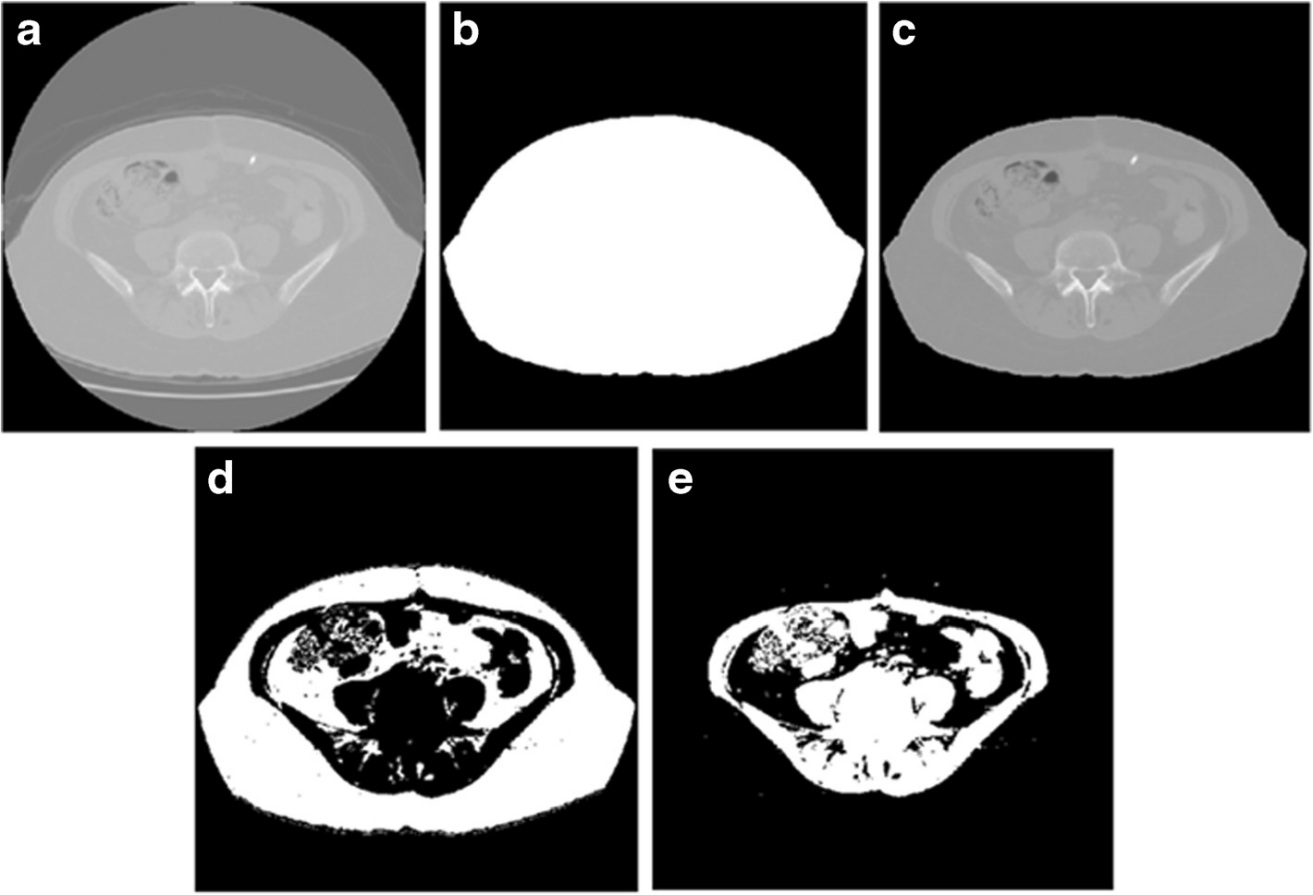
An example of applying our CAD scheme to segment a CT image slice. **a** An original CT image, **b** a CAD-generated body trunk mask, **c** segmented body region, **d** a CAD-generated fat region mask, and **e** a CAD-generated non-fat region mask

First, the scheme was used to detect and segment a body trunk region from the background including air and CT bed depicting on each CT image slice as shown in Fig. 3a. This was done using a previously developed and tested algorithm of automated CT image segmentation [26]. In this method, an operating threshold of −140 HU (determined by previous experiments) is applied to generate a body trunk related mask. Using this threshold, the scheme scans the images from four edges of the CT image slice line-by-line in four different directions namely, from top to bottom, from bottom to top, from left to right, and from right to left, to determine the pixels of the mask boundary. Specifically, in each linear scan, the scheme scans each pixel along the line and keeps moving forward until it hits a pixel, which has a HU value greater than the predetermined threshold. The scheme then defines this pixel as a boundary pixel of a body trunk mask and the scan along this line stops. Once the body trunk mask was defined (as shown in Fig. 3b, it is straightforward to apply this mask on the CT image slice to segment the body section and remove other air background and CT bed sections from the image slice (Fig. 3c).

Second, since CT numbers of the fat pixels range from −140 HU to −40 HU as defined in the previous study [27], these two values are used by the scheme as two operating thresholds to define and segment the body region within the body trunk mask placed on the CT image. The scheme then generates two new masks to cover the fat and non-fat regions as represented by white pixels in Fig. 3d and e, respectively.

**Fig. 4:**
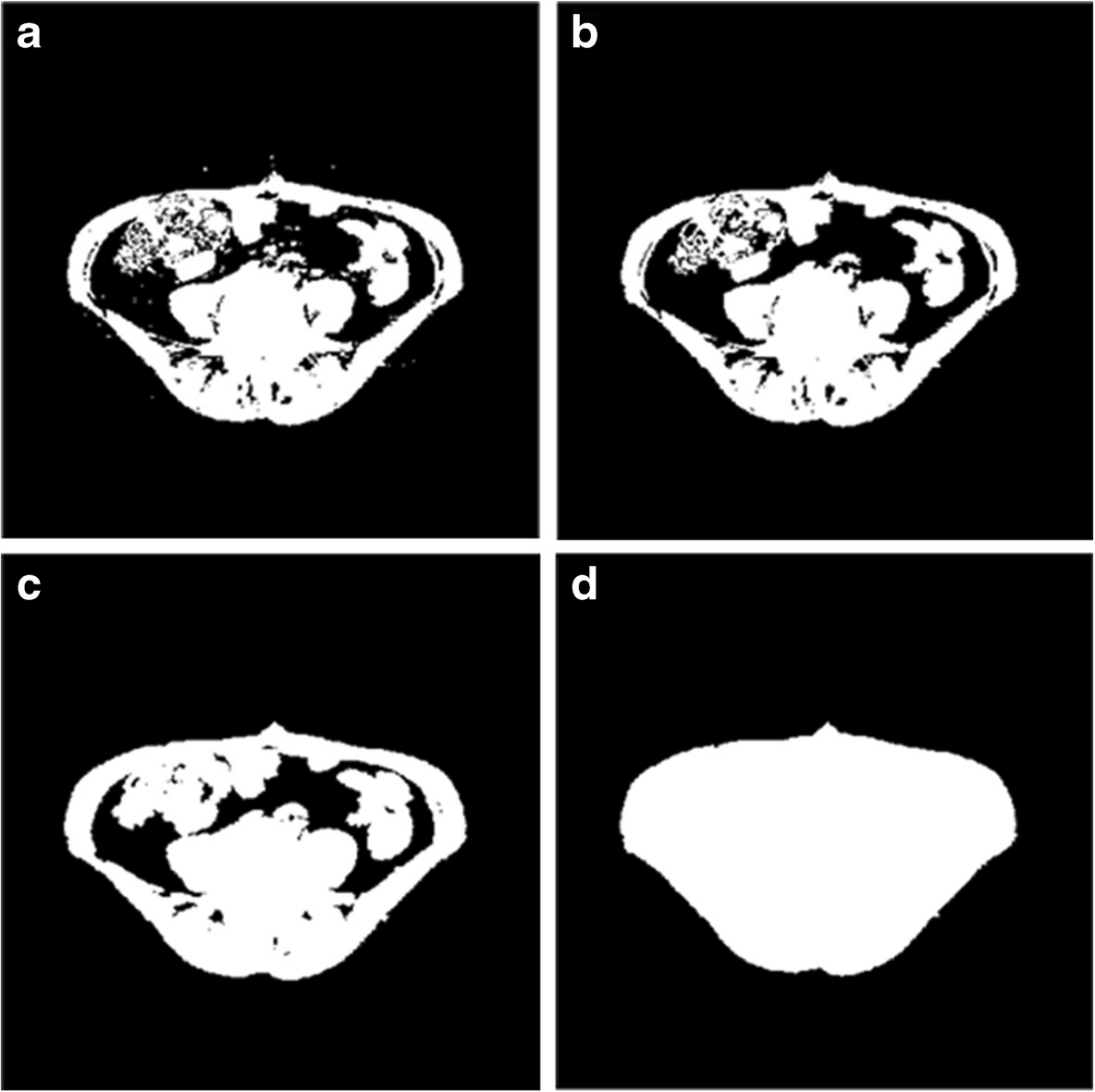
Illustration of defining a visceral region mask, **a** A mask of non-fat area, **b** after removing the small and isolated regions using a pixel labeling algorithm, **c** after a morphological dilation operation, and **d** a mask to cover the entire visceral region

Third, in attempt to detect between VFA and SFA regions, the scheme applies several image processing algorithms to the non-fat region mask and then produces a new visceral region mask [28]. As shown in an example of Fig. 4, a 4-pixel connected labelling algorithm was applied to the non-fat region mask. The scheme removes the connected regions with sizes smaller than a predefined threshold (e.g., 200). As a result, all small and isolated pixels located inside the SFA region were discarded. The scheme then applies a morphological dilation operation with a spherical kernel to the non-fat image region. This process breaks the potential connection between SFA and VFA regions in some CT slices. Last, the scheme creates a visceral region mask by performing a hole-filling algorithm to cover all non-fat structures after a morphological erosion operation (e.g., Fig. 4d).

Fourth, the scheme defines (1) a VFA mask by performing an “AND” logic operator between the fat region mask (e.g., Fig. 3d) and the visceral region mask (e.g., Fig. 4d) and (2) a SFA mask by performing another “AND” logic operator between the fat region mask and body truck mask (e.g., Fig. 3c minus the visceral region mask). After completing the above image processing steps, the scheme is able to classify all image pixels in fat region mask as either the subcutaneous fat pixels or the visceral fat pixels.

Last, these four image processing steps are iteratively performed on all CT slices in the preselected abdominal section of each patient. Our computer-aided scheme was applied to process all 32 cases in our dataset to detect and segment VFA and SFA regions. Although in many previous studies, the manually traced segmentation results were often used as “ground truth” to evaluate accuracy of the automated segmentation, the manually traced boundaries typically suffer from significant inter-reader variability and results in the lower reproducibility [29]. To balance this limitation, we took two approaches and two criteria to evaluate segmentation accuracy of our CAD scheme. First, for each case, SFA and VFA were also manually segmented and measured by a radiologist on one cross-sectional CT image slice visually selected at the umbilicus level using the previous standard method reported in the literature [27]. We then computed the correlation coefficient between the manually segmented SFA/VFA and CAD-segmented/measured SFA/VFA, which is the first evaluation criterion used in this study. Second, since we recognized that the “ground-truth” provided by one radiologist may not be reliable, our ultimate goal is to assess whether we can use quantitative fat or adiposity-related image features that are computed from the automatically segmented VFA and SFA to predict prognosis or clinical outcome of the patients in participation of the targeted chemotherapy. Therefore, our second evaluation criterion is the accuracy of predicting clinical outcomes of the patients using the computed fat image features.

Thus, to predict clinical outcome of the patients, we applied our computer-aided scheme to compute seven image features from the entire segmented CT image slices, which include (1) the ratio (or percentage) of either VFA or SFA volumes as comparing to the whole body volume (size) computed from all scheme-processed CT image slices in the targeted abdominal section, (2) the mean and standard deviation of the CT HU number (pixel value) of the VFA and SFA, and (3) the ratio between the segmented volume between SFA and VFA. In summary, combining with the pre-measured body mass index (BMI) of each patient, we built an image feature pool that includes eight features: ${f}_1$ – BMI, ${f}_2$ – Ratio between SFA and the whole body size; ${f}_3$ – Ratio between VFA and the whole body size; ${f}_4$ – Mean CT number of the segmented SFA volume; ${f}_5$ – Standard deviation of the CT number of all SFA-related pixels; ${f}_6$ – Mean CT number of the segmented VFA volume; and ${f}_7$ – Standard deviation of the CT number of all VFA-related pixels.

Next, we applied logistic regression approaches or models that combine BMI and the computed quantitative features to predict patient’s clinical outcome (PFS or OS). Since a logistic regression based statistical classification model can use or combine two or more continuous variables (or features) to generate binary outcomes that indicate which class the observations (or test samples) belong to [30], we in this study built and optimized two multiple logistic regression classifiers or models to predict PFS and OS of the EOC patients, respectively. For this purpose, PFS and OS values of 32 patients were divided into two classes by using the median PFS and OS value of these 32 patients as the threshold. Two classes then indicate “long” and “short” survival (for both indices of PFS and OS). Specifically, we divided 16 cases into “long” survival and 16 cases into “short” survival classes based on the actually clinical outcome data of these 32 patients. Then, a logistic regression based statistical prediction model was trained and performed to classify these 32 testing cases into these two “long” and “short” survival classes based on the scheme (or model) generated prediction scores.

In order to identify optimal feature set and eliminate un-correlated features to build each model, we applied a Sequential Forward Floating Selection (SFFS) [31] feature subset selection algorithm to identify and select the feature subset with high discriminatory power. In order to minimize the training bias of the logistic regression model, the model performance was trained and tested using a leave-one-case-out (LOCO) based cross-validation method [32]. Specifically, in each model training and testing iteration, the scheme selects 31 cases to train the model and uses one remaining case to test the remaining. SFFS was performed on the training cases to select a subset of features and logistic regression was optimized using these selected features. The evaluation index used in this training and testing process is an area under a receiver operating characteristic (ROC) curve (AUC), which is computed using a maximum likelihood data analysis based ROC curve fitting program (ROCKT, http://metz-roc.uchicago.edu/MetzROC/software, University of Chicago). After building an optimal prediction model, we compared the classification accuracy to a null binomial distribution B(η, ρ) and investigated the significance of the classification performance over a random guess level. Here η is the total observations and ρ is a random guess accuracy (i.e. 0.5). Meanwhile, the classification or prediction accuracy after adding image features was also compared with that using BMI feature only.

## Results

**Fig. 5:**
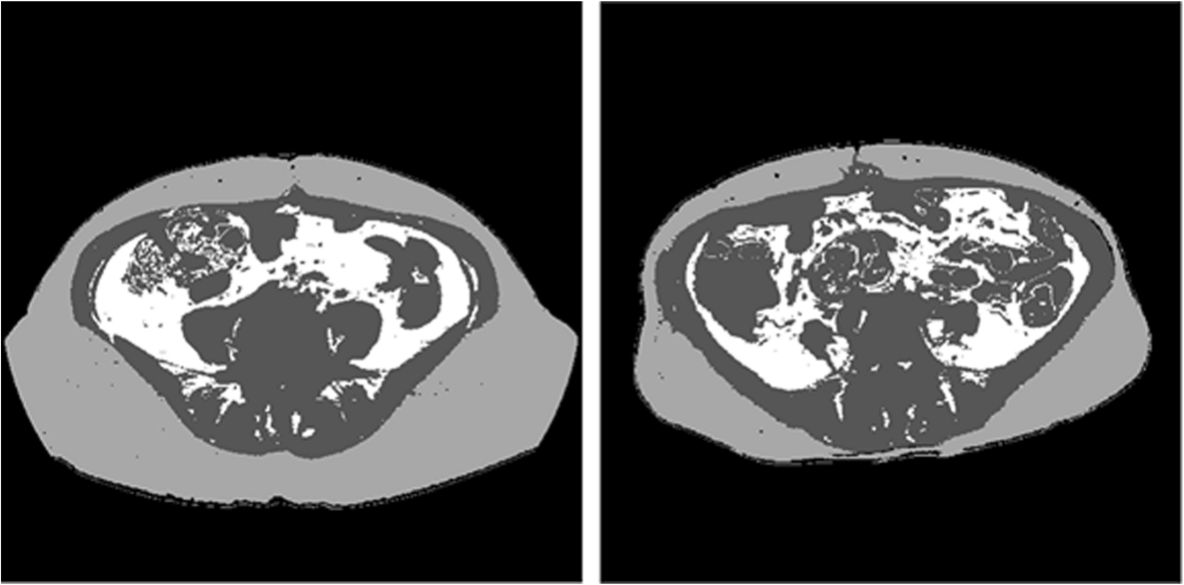
Two examples showing the segmentation of VFA and SFA in four CT image slices. In these two images, SFA is shown in *light gray* color, VFA is represented by *white* color, and *dark gray* color masks other human organs and/or structures

**Fig. 6:**
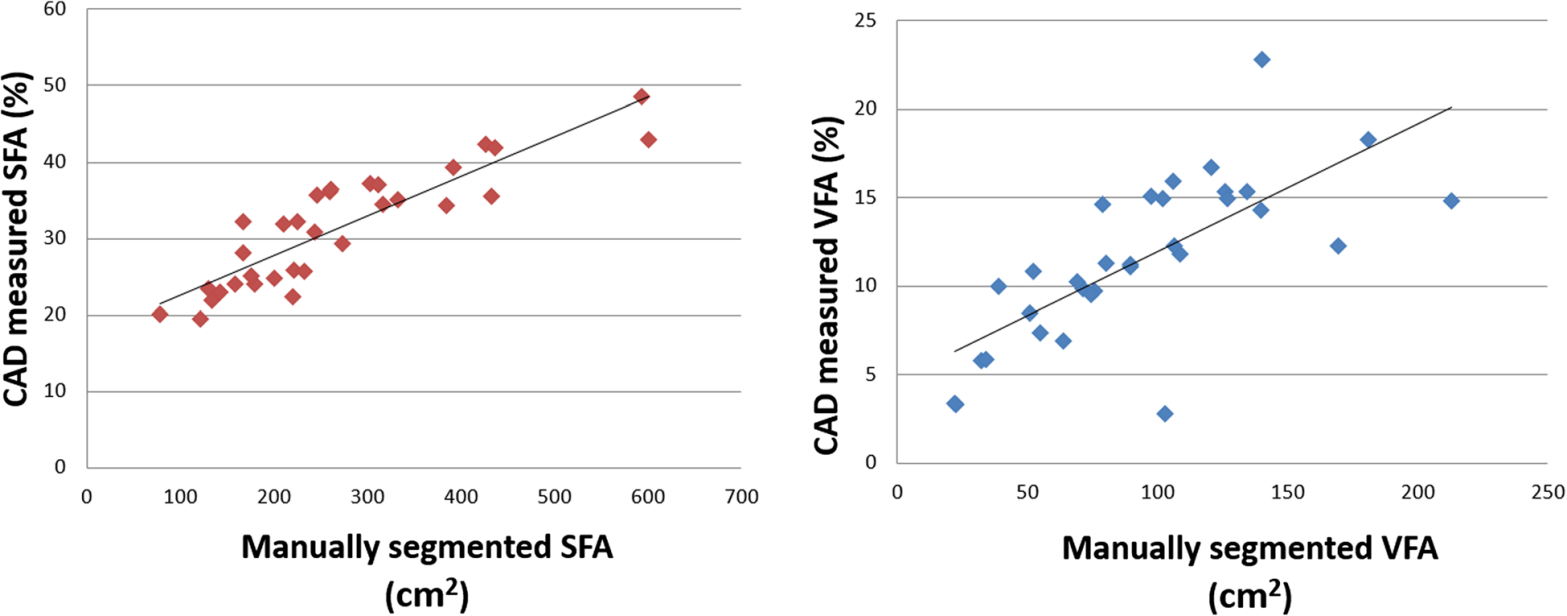
The scatter plot of the manually and automatically segmented SFA or VFA

Figure 5 shows two images that are marked with the segmented VFA and SFA regions depicting on two abdominal CT image slices of interest, which are acquired from two EOC patients in which one patient has substantially higher SFA ratio (a) than another patient (b). Figure 6 shows two scatter plots of the manually segmented SFA/VFA volumes (in cm^2^) and the CAD-segmented/measured SFA/VFA percentage (in %) among all 32 cases in our dataset. The computed correlation coefficients between manual and automated data are 0.886 for SFA and 0.726 for VFA, respectively, which shows a relatively higher correlation of the SFA and VFA segmented between a radiologist and our CAD scheme.

Table 1 summarized the performance of applying the two logistic regression based statistical prediction models to classify the EOC patients in our dataset into two classes of “long” and “short” survival based on the known clinical outcome criteria of both PFS and OS of the patients. It demonstrated that the classification accuracy of PFS was significantly greater (*p* < 0.01) than a binomial null distribution with chance level (i.e. 0.5) accuracy, while the difference is not significant for the logistic regression model used to predict OS. Table 2 ranked the importance of the features for predicting PFS according to their frequencies of being selected by SFFS in the LOO process. It shows that quantitative features were more frequently selected by SFFS and thus possibly more discriminative for predicting PFS than BMI.

**Table 1:** Summary of performance in classifying the two classes of “longer” and “shorter” survival using the multiple logistic regression models

Clinical Outcomes	Prediction accuracy	*p*-value over null hypothesis	AUC	95 % confidence interval
PFS	0.875	9.65 × 10^−6^	0.827	(0.634,0.938)
OS	0.531	0.43	0.505	(0306,0.702)

**Table 2:** Rank of features according to the frequencies of being selected by SFFS for predicting PFS

Feature ID	Frequency of Selection
f_2_	23/32
f_6_	8/32
f_3_	2/32
f_4_	2/32
f_1_	0/32
f_5_	0/32
f_7_	0/32

Table 3 includes and compares two confusion matrixes when using (1) BMI and (2) quantitative features selected by SFFS. When using BMI, 18 of 32 cases were predicted or classified into the correct classes with an overall classification accuracy of 56.3 %. Both positive and negative predictive values are 56.3 % (9/16). After building a logistic regression model using SFFS-selected image features, the overall new model classification accuracy increased to 87.5 % (28/32), which represents a 55.6 % increase as comparing to using BMI only. The results show that the feasibility of using the computed quantitative image features to provide valuable supplementary or complementary information to help increase accuracy in predicting or assessing clinical outcome of the patients treating with chemotherapy.

**Table 3:** Comparison of two computed confusion matrixes between using BMI and SFFS-selected image features to classify patients into two “longer” and “shorter” PFS classes

	BMI		SFFS-selected features	
PFS class	Long	Short	Long	Short
Long	9	7	15	1
Short	7	9	3	13

## Discussion

Identifying quantitative image feature based clinical markers that correlate well with the patients’ clinical outcome is important to establish an optimal personalized cancer treatment strategy and/or develop precision medicine in the future. For this purpose, many studies have been performed by a number of research groups including our group to explore, identify and compute different image feature analysis based clinical markers [13–18]. This study is different from the previous studies in this field (e.g., Radiomics). We demonstrated that the image features computed from non-tumor regions could also provide important and/or supplemental information to assist predicting response of cancer patients to the chemotherapy. Although this study only predicted whether the EOC patients can benefit from receiving the bevacizumab-based chemotherapy, the new computer-aided image processing scheme provides a new quantitative image marker that is also applicable to analyze PFS or OS of EOC patients without receiving maintenance bevacizumab therapy [33] and/or many other different types of cancer patients underwent similar chemotherapy because the angiogenesis and/or vascular endothelial growth factor (VEGF) expression play a fundamental role in the pathogenesis of many types of cancers. Thus, accurately or quantitatively assessment of SFA and VFA is important to determine whether and how the cancer patients should be optimally treated using bevacizumab or other against angiogenesis related chemotherapy.

Since accurate SFA and VFA segmentation is the first step to develop a reliably quantitative image feature analysis approach, which will determine the accuracy of the computed image features as well as the final model prediction results, we developed a simple, computationally efficient and robustly performed scheme to segment SFA and VFA from the volumetric CT image data. Our scheme applies four image processing steps based on the modified region growing algorithms to define a number of corresponding masks that cluster and classify the pixels of each CT image slice into four categories namely, (1) outside the body, (2) SFA, (3) VFA and (4) other human internal organs. We applied this automated image segmentation scheme to all 32 cases in our dataset and visually examined the segmentation results. We did not visually identify any significant segmentation errors in this dataset. We also asked an experienced radiologist to manually segment/trace the SFA and VFA boundary on one CT image of each case using the method reported in the literature [25]. We then computed and compared the correlation coefficients between the manual and automated segmentation results in both SFA and VFA. The relatively higher correlation as shown in Fig. 6 indicates that automated scheme could be used to replace the manual segmentation. Although due to the lack of accurate “ground-truth,” it is often difficult to evaluate the absolute region segmentation accuracy of using an automated scheme, using the computer-aided image segmentation scheme has several advantages to yield high efficient and also avoid inter-reader variability. In another aspect, changes of parameters in the segmentation model only affect the segmentation results of a small percentage of selected CT slices. As a result, measurement from multiple CT slices may provide more robust and accurate results than from a single CT slice.

We also tested the performance of using a number of image features computed from the automatically segmented SFA and VFA regions from CT images to classify the patients into the “long” and “short” survival class groups after receiving the bevacizumab-based chemotherapy. We applied a simple logistic regression based statistical data analysis method to build prediction models and demonstrated that the model-prediction scores have a statistically significant association with the PFS of the EOC patients. It is also quite encouraging to observe from the study results that using the computed SFA and VFA image features yielded substantially higher prediction accuracy than using BMI (as shown in Table 3). Since BMI is computed from height and weight from the patients and is the most commonly used measurement of adiposity in current clinical practices, our results demonstrated that the quantitative features may provide supplementary and useful information other than BMI. This is a more important evidence to support the potentially clinical utility of applying our CAD-based automated SFA and VFA segmentation scheme. Using the CAD scheme, we are able to compute not only the size or volume of SFA and VFA similar to the previous manual method [25], and also other related image features (i.e., the CT number distribution, which relates to the heterogeneity of the SFA and VFA). This is also an importantly potential advantage of developing and applying our CAD scheme.

Despite the promising results, this is a preliminary study with several limitations. First, the dataset size is small and CT images were collected from a single medical institution (or a single CT imaging acquisition protocol). Thus, the 95 % confidence intervals of the AUC values as shown in Table 1 are relatively large. To overcome this issue, further studies and better cross-validation using the new independent datasets are needed before this computer-aided image processing scheme can be accepted and integrated into the advanced quantitative image feature analysis schemes to more accurately predict clinical outcome of EOC patients underwent bevacizumab or other types of chemotherapy. Second, the selection of multiple CT slices belonging to abdomen part was manually processed, which was time-consuming and may introduce inter and intra reader variability. Therefore, further studies will focus on developing automated framework for CT slices selection. Third, due to the limited dataset, the scheme-generated classification scores only significantly associate with PFS, but not OS of EOC patients receiving maintenance bevacizumab based chemotherapy in this study. Further efforts (e.g. extract more features or collect a larger dataset) are required to validate whether OS is significantly related to adiposity characteristics of EOC patients.

## Conclusion

In this study we developed and applied a new computer-aided image segmentation scheme to segment SFA and VFA regions from the volumetric CT image data. We also trained and tested logistic regression models to combine a number of quantitative image features computed from the segmented VFA and SFA regions. Using the leave-one-case-out cross-validation method, our experimental results showed that using this new computer-aided scheme or prediction model enabled to generate a new radiographic image feature marker, which could help more accurately predict which EOC patients are most likely to benefit from receiving maintenance bevacizumab-based chemotherapy than using the traditional BMI based approach. Meanwhile, we also recognized the limitations of this study and identified future research tasks or directions. In summary, we believe that this is a valid technology development study, which demonstrated the feasibility of developing and providing clinical researchers a new computer-aided image processing tool to quantitatively assess a potentially important radiographic image-marker and investigate its association with clinical outcome of cancer patients underwent variety of chemotherapy treatment.
